# Post-myomectomy omental infarction: a case report

**DOI:** 10.1186/s13256-023-03924-y

**Published:** 2023-06-20

**Authors:** Ofunre Eboreime, Godwin Yorwin, Victor Ohenhen

**Affiliations:** 1 Igbinedion University Teaching Hospital, Okada, Edo State Nigeria; 2 Delta State University Teaching Hospital, Oghara, Nigeria; 3 Central Hospital, Benin City, Nigeria

**Keywords:** Omentum, Torsion, Infarction, Myomectomy, Omentoplasty

## Abstract

**Background:**

Omental Infarction (OI) is uncommon and mimics common causes of acute abdomen. It is important to differentiate it from other abdominal conditions that require emergency management. It was first reported in literature in 1896 and about 400 cases have been reported till date.

**Case presentation:**

We reported on a 41 year-old Para 0^+0^ Ibo house wife who presented with 10 years history of supra-pubic mass and five months history of excessive menstrual flow. After physical examination, a diagnosis of symptomatic uterine fibroid was made. She had myomectomy and the raw surface created after the excision of the myomas was covered with omentum. Wound infection developed on the 8th post-operative day leading to a wound breakdown and later partial extrusion of infarcted omental tissue through the dehisced wound. During re-exploration, the infarcted omental tissue was extracted and the residual abdominal abscess was drained. Surgical site wound infection occurred on the 3rd day after re-operation and a sub-acute intestinal obstruction developed on the 4th day thereafter which responded to conservative management.

**Conclusion:**

Careful surgical technique is imperative when utilizing the omentum for reconstructive abdominal surgery. Torsion of the omentum and creation of excess tension while using the omentum for reconstructive procedures should be avoided and increase awareness of this uncommon disease condition by the surgeon is also important. This case is to report a rare finding of omental infarction following myomectomy.

## Background

Omental Infarction (OI) is uncommon and mimics common causes of acute abdomen such as acute appendicitis, cholecystitis, diverticulitis, perforated duodenal ulcer, mesenteric thrombosis and twisted ovarian cyst. [1–5]

OI was first reported in literature in 1896 by Eithel [2, 4, 6–8] and about 400 cases have been reported since then [2]. It occurs more frequently in children and in the 40–50 years age group and more in males than in female in a ratio 2:1 [2].

The incidence of OI is increasing due to the advent of advanced radiological investigations such as ultra-sonographic scan, computerized tomographic (CT) scan, magnetic resonance imaging (MRI) and laparoscopy which are useful tools for diagnosis and in some cases for treatment [3]. Awareness of this disease condition is also on the increase. OI could either be primary (idiopathic) or secondary and can be managed either by conservative or operative methods, which can either be by open or laparoscopic surgery. [1–5]

The aim of this case report was to highlight an uncommon case of secondary omental infarction following the use of omentum to cover the deperitonised portion of the uterus after myomectomy and to caution on its appropriate use by avoiding overstretching, excessive torsion and kinking.

## Case presentation

We reported on a 41 year-old Para 0^+0^ Ibo house wife who presented with 10 years history of a supra-pubic mass which progressively increased in size and five months history of excessive menstrual flow. She could not seek for medical help early enough on account of financial constraints, until her church came to her aid. She was married for 10 years, living intimately with her husband and having adequate sexual intercourse without a conception prior to presentation.

She was a public servant with no history of diabetes or hypertension. Past medical and family histories were not contributory. She neither smoked nor consumed alcohol. She volunteered a history of adverse reaction to chloroquine, amoxycillin and clavulanic acid medications. There was no antecedent history of blood transfusion.

At presentation she looked pale and was afebrile to touch, had a temperature of 36.5 °C, a respiratory rate of 24 cycles per minute, a pulse rate of 84 beats per minute, blood pressure of 130/90 mmHg and a body mass index (BMI) of 20.3. Her packed cell volume was 24%, urinalysis showed significant pyouria. Electrolytes, urea & creatinin and clotting profile were normal. Abdominal sonogram revealed multiple huge uterine fibroids.

The essential findings were in the abdomen. The uterine size was equivalent to 28/52 gestational age and there were no other organomegaly. Vaginal examination did not provide any additional information. A diagnosis of a symptomatic uterine fibroid was made and she was worked up for elective myomectomy after counselling and treated for urinary tract infection with oral ciprofloxacin 500 mg twice daily for five days. She also received three units of blood before surgery.

Prophylactic antibiotics 200 mg of ciprofloxacin and 500 mg metronidazole were also administered intra-venously. Under subarachnoid block anaesthesia she had exploratory laparotomy done through a long midline incision extended above the umbilicus for adequate access. The findings at surgery were a bulky uterus with multiple, widely distributed, varied sized fibroid masses and adenomatous tissues. The uterus was infiltrated with desmopressin 4mcg diluted in 200mls of normal saline and a tourniquet was applied at its base to minimize bleeding. A deperitonised area on the posterior wall of the uterus after myomectomy was covered with omentum sutured around the defect to prevent anticipated bowel adhesions. She received two units of blood intra-operatively.

She developed a fever on the day of surgery through the 5th day post surgery which we attributed to febrile blood transfusion reactions and to malaria fever which is prevalent in our sub-region. Malaria parasites were seen in her blood film. She received appropriate doses of anti-malarial drug, intramuscular injection of artemether 80 mg twice daily for three days anti-histamines, intra-venous promethazine 25 mg stat dose and hydrocortisone 200 mg stat dose. She was discharged home on the 6th day post-operation.

On the 8th post-operative day she presented from home with profuse, purulent and foul smelling discharges from a wound sinus. About 200mls of pus was drained and a wound swab taken for microscopy, culture, and sensitivity. All the stitches were removed and a wick-drain dressing commenced. The wound culture did not yield any growth after three days. On the 36th day post-operation, a blister was noticed in the area of the wound sinus, which ruptured spontaneously and resulted in a wound breakdown three days later.

**Fig. 1:**
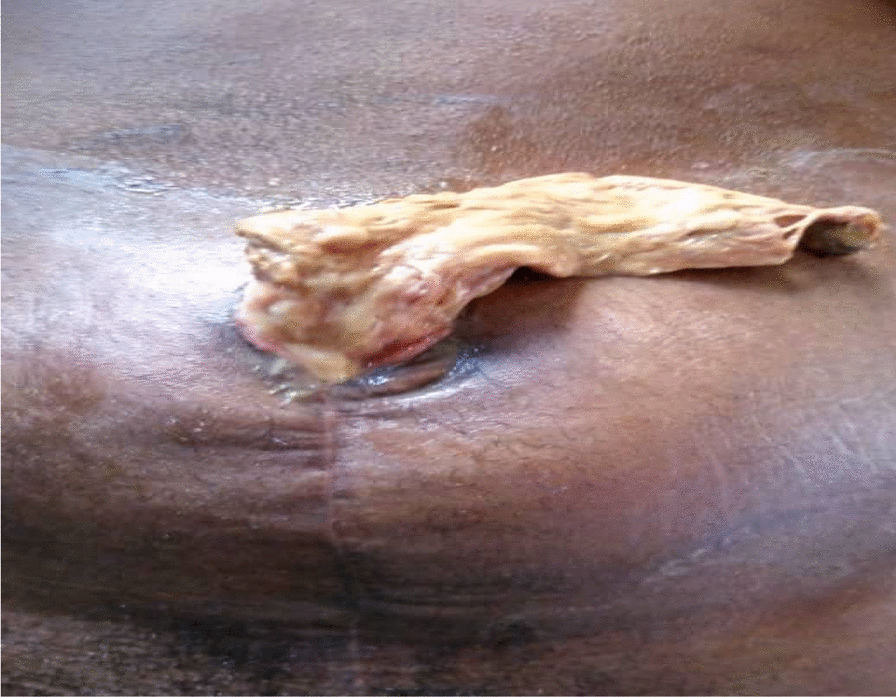
Infarcted omentum extruding from a dehisced abdominal wound

Seven days after the wound break down that is 46th day post-operation, a gangrenous tissue was observed extruding from the dehisced portion of the wound (Fig. 1). On general examination she was calm, not in obvious distress, pale and was not warm to touch. Her temperature was 36 °C, respiratory rate of 20cycles/min, pulse rate of 86/min and blood pressure was 130/80 mmHg. Her packed cell volume was 28% with white cell count of 13,000cells/ml.

The patient was re-admitted, optimised for emergent surgery, rehydrated with three litres of 5% dextrose saline given over four hours, given parenteral anti-biotic therapy of ceftriaxone two grams and metronidazole 500 mg and a unit of blood transfusion. The findings at re-exploration were an infarcted omental tissue partially extruding through the dehisced wound and extending into a pocket of pus on the floor of the pelvis. Other pelvic organs including the uterus were normal. The infarcted tissue was easily and completely extracted leaving a residual cavity full of pus and the infarcted tissue was sent for histology. A copious saline lavage of the cavity was done and a drain was left in the residual cavity. The abdominal wound was closed in a single layer. Parenteral broad spectrum anti-biotics which were used for prophylaxis were continued post-operatively. Surgical site wound infection occurred on the third day of post re-operation.

A day later, patient developed signs and symptoms of partial intestinal obstruction which was confirmed by abdominal radiography. The obstruction resolved after four days on conservative management which involved nil per mouth, intravenous infusion of 500mls 5% dextrose saline administered four-hourly, placement of nasogastric tube and the continuation of the parenteral antibiotics. The patient was eventually discharged home on the 11th day of re-admission. A four-month follow up was uneventful.

This case is to report a rare finding of omental infarction following a myomectomy.

## Discussion

Omental infarction is not a common disease condition and since it was first described in 1896, only about 400 cases have been described till date [2, 10]. OI accounts for 7% of all cases of acute abdomen visiting the accident and emergency [2] and about 0.1% of laparotomies done for emergent abdominal conditions [1]. The accuracy of pre-operative diagnosis of OI is about 0.6–4.8% [10].

OI can either be primary (idiopathic) or secondary. The unnecessary length of omentum, its tenuous vasculature, increased abdominal pressure and increased peristalsis from over eating are some of the patho-physiological mechanisms involved in idiopathic omental infarction (IOI) [1].

Precipitating factors in IOI include obesity, 40–50 age group, male gender, exertion, local trauma, occupational vibration, excessive straining and coughing and sudden change in posture [1, 2]. Omental pathologies like cysts, tumour invasion, omental malformations like bifid omentum, vascular variant, kinking of the omentum secondary to congenital attachment abnormalities, local variation in fat deposition, and redundant omental vein also play a role in this condition [2, 3, 6, 8, 10]. Other causes of IOI are hyper-coagulability, congestive cardiac failure, superior mesenteric artery/venous thrombosis and vasculitis [6].

Secondary causes of OI include strangulated inguinal hernia, intra-abdominal tumours, pelvic inflammatory disease, post-operative venous thrombosis, arterial ligation and kinking from adhesions from previous abdominal surgery [1, 6]. Secondary OI has been associated with colonic resections, gastrectomies, and pelvic surgeries [6]. In this index case secondary omental infarction occurred after a length of the omentum was used to cover a denuded portion of the uterus after myomectomy on a huge uterine fibroid probably resulting from overstretching, kinking, excessive tension or from post-operative adhesions.

On account of the proclivity of the omentum for adhesion formation, neovascularisation, defence against infection and its abundant availability in length, it is used in the reconstruction of defects in abdominal and pelvic surgeries [2]. These properties of the omentum prompted us to use the omentum to cover an area of the uterus devoid of peritoneal covering after the removal of the fibroid masses.

The right side of the omentum is more involved than the left on account of its longer length and more mobility and for this reason the resultant abdominal pain is often right sided in 90% of cases [1, 3, 5, 11].

In the presence of predisposing and initiating factors, torsion of the omentum occurs along its long axis and on its narrow pedicle causing the kinking of the veins and arteries leading to venous stasis, thrombosis, haemorrhagic infarction and finally fat cells necrosis [1, 10]. OI may ultimately result in the formation of a fibrous ball and adhesions leading to the formation of loose ball inside the abdominal cavity or when it becomes infected results in an abscess [10]. In our case, omental infarction was followed by infection and abscess formation in the pouch of Douglas.

Abdominal ultrasonography (USS) and computed tomography (CT) scan are useful tools in the pre-operative diagnosis of OI [3, 4, 12] and are vital in considering conservative option of management which is frequently successful [5]. The use of these radiologic tools has increased the diagnosis of OI [6]. They also help to exclude other pathologies like omental deposits and tumours [6, 8]. CT scan shows characteristic appearances of twisted omentum like caking, hazy stranding, nodules, signs of whirl fatty mass formation with concentric linear strands and diffuse infiltration [2, 7, 8, 12, 13] These features may mimic recurrent abdominal malignancy [9]. When diagnosis by radiological imaging fails, laparoscopy is then the next option for definitive diagnosis and perhaps for treatment [5]. In this case diagnosis was essentially clinical hence there was no paramount need for further radiological investigations.

Histological features of OI consists of focal necrosis, and fibroblastic reactions [3]. These were essentially similar to the histological findings in our study. In this case presentation, there was no complaint of abdominal pain, nausea and vomiting. In other studies too, symptoms like nausea, vomiting, fever and diarrhoea were infrequently encountered [6, 7]. It is probable that the pain during the process of torsion of the omentum in this case was subsumed into the normal post operative pain or perhaps the torsion was gradual in onset.

There was leucocytosis and anaemia in this case similar to other studies [6] probably due to omental infarction, peritoneal abscess formation and sepsis.

This case had exploratory laparotomy, removal of the infarcted tissue and drainage of the abscess collection in the peritoneal cavity unlike some cases of idiopathic omental infarction where treatment is often conservative. The option of conservative management requires confirmation and follow up with ultra-sonographic or CT scan. Failure of expectant management may lead to definite treatment by open omentectomy or by laparoscopic excision of the infarcted omentum [1, 4, 5]. Indications for surgery include failed conservative management, severe pain, adhesions, intestinal obstruction, abscess formation and persistent fever [1, 6].

The omentum is useful in reconstruction in abdominal and pelvic surgery [2]. In our case, the raw surface created after myomectomy was covered with an omental patch. An iatrogenic twist perhaps occurred during the process of omentoplasty or a torsion may have resulted from subsequent adhesions probably facilitated by the injection of desmopressin.

## Conclusion

Omental infarction is a rare disease and may mimic common causes of acute abdomen such as acute appendicitis, cholecystitis, diverticulitis, perforated duodenal ulcer, mesenteric thrombosis and twisted ovarian cyst. Secondary omental infarction may result from the use of the omentum for reconstruction in pelvic surgery such as after myomectomy. When the omentum is used for reconstructive surgery, utmost care must be taken in the handling of the omentum, ensuring that tension, twisting and kinking are avoided. It is worth noting that OI has a favourable prognosis when properly managed. Surgeons' awareness of OI, the use CT scan and laparoscopic approach to management is imperative [10]. OI has a favourable prognosis with early diagnosis and appropriate management.

## Data Availability

Not applicable.

## Abbreviations

OI: Omental Infarction
IOI: Idiopathatic omental infarction
USS: Abdominal ultrasonography
CT: Computed tomography
MRI: Magnetic resonance imaging
Para: Parity
28/52: 28 weeks
mg: Milligram
mmHg: Millimetre mercury
